# The practice of psychological well-being education model for poor university students from the perspective of positive psychology

**DOI:** 10.3389/fpsyg.2022.951668

**Published:** 2022-08-01

**Authors:** Ling Luo

**Affiliations:** Students’ Affairs Department, Henan Polytechnic University, Jiaozuo, China

**Keywords:** poor college students, mental health education, BP neural network algorithm, fuzzy comprehensive evaluation, psychological prediction

## Abstract

Poor university students are a special group. Social development provides many positive factors for poor university students’ personality and psychological development, but negative factors are also accompanied by them, which affect the psychological health of poor university students. University students are in a period of rapid physical and mental development, and it is an important issue that colleges and universities need to solve psychological well-being education. We hope to find out the aspects that can be studied in the irregularity of various factors that affect college students’ mental health. BP neural network is a typical model of artificial neural network. Based on the BP algorithm and the fuzzy comprehensive evaluation of the psychological well-being prediction system for poor university students, this paper systematically summarizes the concept of psychological well-being, the factors that affect psychological well-being, and the related research done by predecessors on psychological well-being. Using the international psychological well-being scale SCL-90 to comprehensively consider the psychological well-being status of poor university students and select the optimized BP algorithm to establish a psychological well-being prediction model, and implement it and compare it with other models to reflect its superiority. Data were collected and analyzed by means of a questionnaire. The regression model was used to analyze the relationship between mindfulness, rumination and psychological well-being. The mediation index fitted by the model reached 0.9. The model can reflect the real situation of the data, that is, rumination plays a partial mediating role in the effect of mindfulness on psychological well-being. The introduction of this psychological prediction model into the psychological well-being education of poor university students not only helps to improve the educational concept and expand the educational approach, but also helps to achieve the goal of psychological well-being education for poor university students, thereby promoting the improvement of the psychological quality of poor university students.

## Introduction

In this era of rapid knowledge and economic development, the society’s requirements for high-quality talents are increasing day by day. Student groups are the representatives of high-level talents. For the main influencing factors of psychological well-being, there are certain differences between university students and other groups, which induces their mental illness. The reason is also fundamentally different from other groups. The research on the psychological problems of university students should take into account that the era of university students and the contemporary cultural background directly affect the psychological well-being of university students. Work brings great stress. With the fierce competition in today’s society and the increasing pressure of life, the psychological well-being problems of university students are becoming more and more prominent, and the psychological well-being status is worrying (Waters et al., 2022). Among the university students, there are many people who are forced to suspend school, drop out, self-mutilate, commit suicide, or even commit crimes due to serious psychological disorders or psychopaths. Whether the psychological well-being of university students is affected by many factors, and there are inextricable links between each factor, so the prediction of university students’ psychological well-being is a non-linear problem (Zhen, 2017). And poor university students are a special group. In this paper, the neural network algorithm based on BP algorithm and fuzzy comprehensive evaluation model are used to solve the problem of psychological health prediction of poor university students. Its functions not only have associative memory, non-linear mapping, classification, and recognition, but also have intelligent features such as optimal calculation and knowledge processing. Because these unique capabilities happen to solve the problem of psychological well-being prediction, which is inherently non-linear mapping.

Existing research on the psychological well-being level of poor university students, most scholars use the SCL-90 symptom self-rating scale (Lejiao, 2018), and use two independent samples *t*-test to compare with non-poor groups. The results show that there are significant differences between poor university students and non-poor university students in depression, anxiety, somatization, strong pursuit symptoms, interpersonal relationships, and terror, as well as the overall average scores; these indicators of terror are significantly different from the norm of university students (Li, 2020; Abdolrezapour and Ghanbari, 2021). In the above results, impoverished university students are more negative, which also shows that the psychological well-being level of impoverished university students is worse than that of non-poor university students. Two independent samples *t*-tests were conducted on the basis of gender among poor university students. The results showed that females had significant differences with males in somatization, depression, anxiety, hostility, terror, and overall average scores. Females were more negative, especially compared with men, the scores of the two indicators of interpersonal sensitivity and depression are significantly different. Scholars speculate that this may be related to the unique personality characteristics of women. At the same time, they also found that poor university students whose parents both go out to work are more paranoid and mentally ill than their parents. Poor university students who go out have high scores (Shi and Yuan, 2017). In addition, studies have shown that university students from poor families have higher scores on other factors than somatization and obsessive-compulsive symptoms than university students from non-poor families. It is speculated that the reason is the probability of negative life events suffered by university students from poor families, much larger than that of university students from non-poor families.

Mindfulness is a favorable means to improve psychological well-being, while rumination is an unfavorable factor affecting psychological well-being. There is a certain relationship between the two in terms of their connotations. Mindfulness can play a certain role in intervening in rumination. On the theoretical side, the concepts of rumination and mindfulness are supported by the data in the text and different research conclusions. In addition, research on mindfulness, rumination and psychological well-being will help to analyze the relationship between the three, and to explore the internal mechanism of the effects of mindfulness and rumination on psychological well-being, which can enrich the research results of rumination and mindfulness. The research results will provide a reference analysis for the psychological well-being education of poor university students.

The innovation of this paper is to understand the relationship between mindfulness and rumination and the mental health of poor college students through predictive analysis, so as to provide reference for the mental health education strategies of poor college students. Based on BP neural network algorithm and fuzzy comprehensive evaluation model, this paper investigates the mental health development of this group by using the internationally recognized SCL-90 scale and questionnaire survey method. Introducing this psychological prediction model into the mental health education of poor college students will not only help to improve the educational concept and expand the educational approach. It also helps to achieve the goal of poor college students’ mental health education, so as to promote the improvement of poor college students’ psychological quality.

The chapter arrangement of this paper: Chapter 1 introduces the concept of positive psychology in psychology and related research on psychological prediction; Chapter 2 introduces the psychological prediction algorithm based on BP neural network model; Chapter 3 data analysis on the psychology of poor university students Health makes prediction analysis experiments; Chapter 4 summarizes the relationship between mindfulness and rumination on psychological well-being based on the prediction analysis model; Chapter 5 summarizes the full text.

## Related work

In the field of psychology, positive psychology has become a trend in the field of psychology (Guo and Long, 2019). The Positive Psychology Society has been established in the United States, Europe and other places, and regularly holds annual research conferences on positive psychology in these regions every year, and holds a world positive psychology summit every 2 years. At present, positive psychology mainly focuses on subjective well-being, with positive experience, positive personality, and positive social system as the three supporting points to carry out related research, and has formed a basically complete set of theoretical systems.

Xiaojie et al. (2018) proposed that positive experiences are mainly individual experiences, which can be divided into two categories: positive emotions and positive emotions. Positive emotions refer to the subjective feelings of individuals, such as: love, satisfaction, pleasure, satisfaction, excitement, etc. Positive emotions refer to the objective feelings of individuals, such as love, happiness, optimism, ideals, self-improvement, etc. These experiences can be individual present feelings, tastes of the past and aspirations for the future. In addition, positive psychology believes that individual satisfaction and happiness in the past and hope for the future are important conditions for positive experience (Xiaojie et al., 2018). Zhiyong et al. proposed that positive psychology focuses on the development of positive personal traits of individuals. The main research includes 24 positive personal traits, which basically belong to the category of personality traits, such as: good temperament, noble virtues, correct values, elegant interests, Outstanding abilities, gratifying achievements, physical and psychological well-being, good interpersonal relationships, etc. Positive personality traits are actually the development of an individual’s potential in real life. When a certain potential ability is activated, a benign behavior is formed, which promotes the formation of positive personal characteristics. Therefore, activating self-potential is a method of cultivating positive personal characteristics (Zhiyong et al., 2017). Psychology has three major missions: research on negative psychology to treat mental illness, make everyone’s life more meaningful and meaningful, and identify and cultivate geniuses (Wang et al., 2021). Existing research on the psychological well-being level of poor university students, Gong et al. uses the SCL-90 symptom self-rating scale, and uses two independent samples *t*-test to compare the group based on whether it is poor. Anxiety, somatization, obsessive-compulsive symptoms, interpersonal relationships, fear, and their overall average scores are significantly different from those of university students without poverty. The norm was significantly different. In the above results, poor university students are more negative, which also shows that the psychological well-being of poor university students is worse than that of non-poor university students (Gong and Jiang, 2018). Poor university students have significant differences in negative emotions, positive coping styles, self-esteem and interpersonal relationships with non-poor university students; poor university students during the poverty period, the younger the age at the beginning of poverty, the longer the time of poverty, parents and children. The lower the frequency of contact, the higher the level of depression and anxiety in adulthood, the lower the level of self-esteem, the more interpersonal troubles, and the less positive coping styles are used. Compared with non-poor university students, poor university students have lower social support (Bocheliuk et al., 2021). Zhang and University (2017) found that the health status of non-poor children was better than that of poor children and poor children. Among poor children, poor children of the same age or without caregivers had the worst psychological well-being, and children cared by a single parent were better than other poor children. In addition, the scores of interpersonal sensitivity and spiritual quality of poor university students are higher than those of non-poor university students, and they are more inclined to be introverted (Zhang and University, 2017). Wang et al. (2017) believes that mindfulness is actually an ideology of continuous perception of what is happening to the individual and the stimulation of the moment. Chu and Yin (2021) used multiple linear regression and path analysis methods to explore the influencing factors and mechanism of university students’ inhibition. Zhang et al. (2017) established a mathematical model of the psychological well-being prediction system of university students based on neural network, which provided the basis for the prediction of psychological well-being to a certain extent. Jiang (2020) uses neural network and fuzzy mathematics to establish a mathematical model to evaluate psychological well-being status, and the verification results are good. Some scholars believe that psychological well-being is a state of complete well-being or completeness of the mind. Its essence believes that psychological well-being is a continuous psychological state, in which an individual has the vitality of life, positive inner experience, good social adaptation, and can effectively exert one’s physical and mental potential and positive social ability (Yurayat and Seechaliao, 2021). Chen (2019) designed a design scheme of a data mining system for university students’ psychological well-being problems. The main method is to use the ID3 algorithm to build a decision tree for classification mining, and use the Apriori algorithm to mine association rules. From this article, it can be seen that there is a good background in psychological research, but the data used are only obtained from the psychological well-being test questionnaire, and the research objects are only students of a certain grade in the author’s school (Chen, 2019). Han (2020) uses the decision tree C4.5 algorithm to build a psychological well-being assessment model, constructs a decision tree, and predicts psychological well-being by extracting rules. The literature only uses a relatively simple classification algorithm for applied research, and does not involve the analysis of the factors associated with the formation of university students’ psychological well-being problems (Han, 2020). Wang (2020) applied a tree mining algorithm called IMB3-Miner to obtain knowledge and patterns from semi-structured mental illness patient record data to explore the influence of genetic and environmental factors on psychological well-being problems to provide insights for the prevention and treatment of mental illnesses Useful information (Wang, 2020).

The study of positive psychological well-being education for university students is a work aimed at improving happiness, developing self-potential and cultivating positive qualities. It is different from traditional psychology, which only focuses on the negative aspects of food. It focuses on negative aspects. At the same time, it pays more attention to the individual’s own inner strength and good quality. And through the cultivation of inner strength and good quality to promote the healthy growth of individuals and adapt to the needs of future social development (Spinazze et al., 2019; Gokten and Uyulan, 2021). This is an indispensable educational activity in the education and teaching work of university students. It stimulates the positive potential of university students by strengthening their own inner strength, and combines the psychological needs of university students to put forward educational concepts and training models. According to the development of university students’ future life, education and training Intensive learning to improve inner potential and strength. Through social practice and quality extension training, we can improve university students’ cognition of the future society, ensure the improvement of university students’ abilities, and lay a good psychological foundation for university students’ future life development.

## Psychological evaluation model based on BP neural network algorithm and fuzzy comprehensive evaluation model

Based on the international psychological well-being scale SCL-90, this paper comprehensively considers the psychological well-being status of poor university students, and uses BP neural network algorithm and fuzzy comprehensive evaluation model to establish a psychological well-being evaluation model.

**FIGURE 1:**
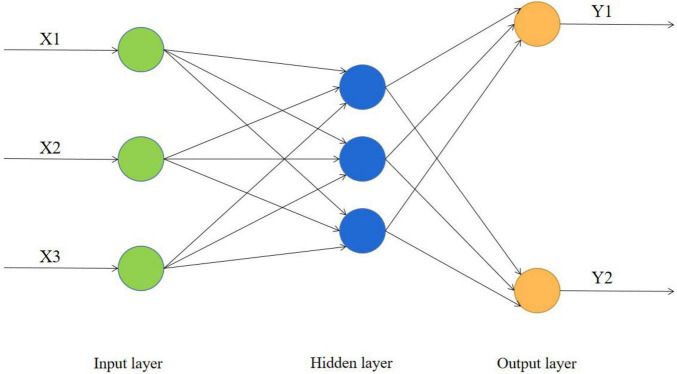
BP neural network algorithm framework.

The basic idea of the BP algorithm is to modify the network weights and thresholds to make the error function descend along the gradient direction. In this network, each processing unit has a non-linear input and output relationship, and its function usually adopts the sigmoid function. The learning process of the BP network is divided into two parts: forward propagation and error back propagation: in the first stage, the input sample passes through the input layer, is processed layer by layer through the hidden layer and calculates the actual output value of each unit; in the second stage, the actual output value of each unit is calculated, if there is an error between the actual output of the output layer and the expected output value, the difference between the actual output and the expected output is calculated recursively layer by layer, and this error is the basis for correcting the weights of neurons in each layer. The process of continuously adjusting the weights is the learning and training process of the network, until the error reaches the expected error, the network learning process ends (LaBrie et al., 2015). The BP neural network framework is shown in Figure 1.

The action function adopts the unipolar Sigmoid function as shown in Formula (1).


(1) $$\left[f\,\left(x\right)=\sqrt{\frac{1-e^{x}}{1+e^{-x}}}\right]$$

For the input vector *^X^*, the input of the output layer node*^k^* of the neural network is shown in *^net^*^_k_^ Formula (2).


(2) $$n\,e\,t_{\text{k}}=\sum_{j=0}^{m}w_{\text{jk}}\,y_{\text{i}}$$

The actual output ^*O*_*k*_^ of the output layer node*^k^* is shown in Formula (3).


(3) $$O_{\text{k}}=f\,\left(n\,e\,t_{\text{k}}\right)$$

The input *^net^*^_*j*_^ of the hidden layer node *^j^* is shown in Formula (4).


(4) $$n\,e\,t_{\text{j}}=\sum_{i=0}^{n}v_{i\,j}\,x_{\text{i}}$$

The actual output ^*y*_*j*_^ of the hidden layer node *^j^* is shown in Formula (5).


(5) $$y_{\text{j}}=f\,\left(n\,e\,t_{\text{j}}\right)$$

When the actual output of the BP network is not equal to the expected output, there is an output error, which is defined as shown in Formula (6).


(6) $$E=\frac{1}{2}\,\sum_{k=1}^{l}{\left(d_{\text{k}}-O_{\text{k}}\right)}^{2}$$

Expand the above output error definition to the hidden layer as shown in Formula (7).


(7) $$E=\frac{1}{2}\,\sum_{k=1}^{l}{\left(d_{\text{k}}-f\,\left(n\,e\,t_{\text{k}}\right)\right)}^{2}=\frac{1}{2}\,\sum_{k=1}^{l}{\left(d_{\text{k}}-f\,\left(\sum_{j=0}^{m}w_{i\,j}\,y_{\text{i}}\right)\right)}^{2}$$

The adjustment of the weights is precisely to make the error decrease continuously, so the adjustment of the weights should be proportional to the decrease of the error, as shown in Formula (8).


(8) $$\Delta \,w_{i\,j}=-\eta \,\frac{\partial E}{\partial w_{\text{jk}}}$$

Assign random numbers to the weights of the nodes in the hidden layer and the output layer, and set the learning rate ^∂ ∈ (0, 1)^ and the expected error. A training set sample is selected from the training mode set, and its output mode and expected output are sent to the network (LaBrie et al., 2015; Chen and University, 2018). BP neural network is one of the most widely used neural network models, but it still has limitations, in order to better apply it to solve practical problems. The BP network realizes a mapping function from input to output, and in mathematical theory, it has been proved to have the function of realizing any complex non-linear mapping. Having this feature makes it ideal for solving complex problems. The trained BP network runs very fast and can be used for real-time processing. BP network has instability in the process of learning and memory. The newly added samples will affect the learned samples. At present, this phenomenon is inherent in the BP network and cannot be avoided. Therefore, the original learning samples and the newly added learning samples must be put together to retrain the network.

In applying the BP network in practice, it is often difficult for this paper to determine an optimal learning rate that is suitable from the beginning to the end. Generally speaking, when the error surface is flat, it is desirable to increase ^η^ to speed up the convergence; and when the error surface changes drastically, it is desirable to decrease ^η^o prevent over-learning from increasing the error. Therefore, in order to speed up the convergence of the BP algorithm, a good idea is to adapt the learning rate dynamically, that is, to adjust the learning rate dynamically. Its adjustment formula is shown in Formula (9).


(9) $$\eta \,\left(k+1\right)\,\left\{ \begin{array}{c} 1.05\,\left(k\right),E\,\left(k+1\right)<E\,\left(k\right)\\ 0.7\,\left(k\right),E\,\left(k+1\right)>1.04\,E\,\left(k\right)\\ \eta \,\left(k\right),o\,t\,h\,e\,r \end{array}\right.$$

Among them, *^E^*^(*k*)^ is the *^k^*-th network error sum of squares, and ^η(*k*)^ is the *^k^*-th network learning rate.

When selecting the number of neurons in the input layer of the BP network, the variables that can represent the overall characteristics of the sample should be appropriately selected from the actual situation. In general, two basic principles must be followed: those variables that have a large impact on the output and can be detected or extracted must be selected. There cannot be a very strong linear relationship between the selected input variables (Lijuan, 2016).

## Psychological well-being prediction of poor university students

### Influencing factors of psychological well-being of poor university students

The psychological well-being level of university students is affected by many factors. Among the many factors that affect the psychological well-being of university students, there are not only internal factors such as personal physiology and psychology, but also external factors such as adverse factors in the social environment. With the continuous increase of enrollment in colleges and universities, the number of university students has greatly increased, which has led to the lack of balanced distribution of educational resources and the difficulty of finding employment after graduation. This causes the gap between the real life and the ideal of university students is too large, which is a root cause of the prominent psychological problems of university students (Cheng and Yan, 2016). Shaping the positive qualities of individuals is the primary goal of current psychological well-being education.

Internal factors, that is, psychological factors, are the main factors leading to student suicide. The survey results show that the psychological factors that affect psychological well-being and lead to the occurrence of mental diseases are introverted, pessimistic and sad, fragile will, and lack of awareness of self-worth. Students with these “diseases” are very likely to commit suicide when they encounter adverse stimuli (Jeon and Oh, 2017).

External factors, namely social factors, are complex and multifaceted. Interpersonal disputes, that is, many problems encountered in dealing with the surrounding environment and interpersonal communication, such as: conflicts with parents, tension and conflict in social interpersonal relationships, etc., which also lead to suicidal behavior. At the same time, with the gradual increase in the number of young students falling in love, emotional entanglement leading to lovelorn has also become one of the important reasons for suicidal behavior. In addition, the influence of bad culture and various mental illnesses are also important aspects that cannot be ignored.

### Data collection

There are a total of nine factors in the internationally used SCL-90 scale, which are used as part of the factor set for judging the model. This paper comprehensively considers the psychological well-being status of poor university students, and regards family composition and whether they are an only child as the factors related to the judgment. Therefore, the factor set of the evaluation model for the psychological well-being status of poor university students studied in this paper is due to the items contained in each factor. There are 12 items in F1, 10 in F2, 9 in F3, 13 in F4, 10 in F5, 6 in F6, 7 in F7, and 6 in F8. There are 10 items for the F9 factor, and 7 items for other items that are not included in the above factors, which are listed as “other.” As shown in Table 1.

**TABLE 1:** Average score of SCL-90 scale factors.

Factor set	SCL-90 corresponding question number	Representation
Somatization (12 items)	1, 4, 12, 27, 40, 42, 48, 49, 52, 53, 56, 58	F1
Obsessive-compulsive symptoms (10 items)	3,9, 10, 28, 35, 45, 46, 51, 55, 65	F2
Interpersonal sensitivity (9 items)	6, 21, 34, 36, 37, 41, 61, 69, 73	F3
Melancholy (13 items)	5, 14, 15, 20, 22, 26, 29, 30, 31, 32, 54, 71, 79	F4
Anxiety (10 items)	2, 17, 23, 33, 39, 57, 72, 78, 80, 86	F5
Hostile (six items)	11, 24, 63, 67, 74, 81	F6
Horror (seven items)	13, 25, 47, 50, 70, 75, 82	F7
Paranoia (six items)	8, 18, 43, 68, 76, 83	F8
Psychotic (10 items)	7, 16, 35, 62, 77, 84, 85, 87, 88, 90	F9

**FIGURE 2:**
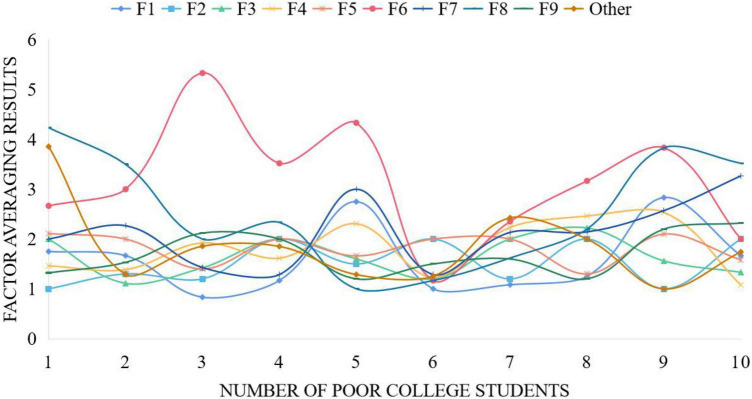
Some sample data processing results.

This paper collects data in the form of questionnaire survey, distributes questionnaires to impoverished university students in a university, collects SCL-90 scale data, and uses BP data network algorithm to perform factor averaging. The results after averaging are shown in Figure 2. It can be seen that the treatment result of factor 6 is the highest among the average results of factors. At the same time, the treatment result of factor 1 is relatively low. With the increase of the sample number of poor college students, the average results of different factors tend to be consistent.

### Multi-level fuzzy comprehensive evaluation model

By analyzing each factor in the factor set, the factors of the same category are classified into one category. In this way, the factor set can be divided into 4 categories, and each category contains three factors. As shown in Tables 2, 3.

**TABLE 2:** Classification of factor sets.

Category	Representation
Physical characteristics of poor university students	G1
Poor university students interact with people externally	G2
The inner character and emotion of poor university students	G3
Basic information of poor university students	G4

**TABLE 3:** Factor set representation.

Category	Factor set	Representation
Physical characteristics of poor university students	Somatization	F1
	Anxiety	F2
	Psychotic	F3
Poor university students interact with people externally	Interpersonal sensitivity	F4
	Hostility	F5
	Fear	F6
The inner character and emotion of poor university students	Obsessive-compulsive symptoms	F7
	Melancholy	F8
	Paranoid	F9
Basic information of poor university students	Account type	F10
	Family composition	F11
	Is it an only child	F12

It is of great significance to effectively and scientifically evaluate the identification of the poverty level of college students. The work of identifying the poverty level of college students has the characteristics of uniqueness, complexity and fuzziness. Using fuzzy comprehensive evaluation method to identify the poverty level of college students, the method is simple, feasible, and can scientifically and truly reflect the actual situation of identifying the poverty level of college students, which has a certain practical significance. Several factors should be considered for the degree of family poverty of an evaluation object. The commonly used indicators are: monthly living expenses of the applicant, regional factors of the health status of the whole family, family income, sudden disasters suffered by the family, etc. This paper conducts three memory less iterative statistical experiments on several poor university students, and uses the fuzzy comprehensive evaluation method to assign weights. The established secondary evaluation matrix is shown in Formula (10).


(10) $$G\,1=\left[\begin{array}{c} F\,1\\ F\,5\\ F\,9 \end{array}\right],G\,2=\left[\begin{array}{c} F\,3\\ F\,6\\ F\,7 \end{array}\right],G\,3=\left[\begin{array}{c} F\,2\\ F\,4\\ F\,8 \end{array}\right],G\,4=\left[\begin{array}{c} F\,10\\ F\,11\\ F\,12 \end{array}\right]$$

Select the most important *^q^* subsets in the factor set, that is, select ^*p*_1_^ = ^*q*^ factors in the *^G^*, as shown in Formula (11).


(11) $$G^{\left(j\right)}=\left\{G_{\text{i}}^{\left(i\right)}\right\}⊃G_{s-1}^{\left(i\right)}$$

The weights are established for the first-level factor set, that is, the weight distribution of each factor in the factor *^G^* = ^{*G*1,*G*2,*G*3,*G*4}^ set. Comprehensive evaluation is performed on each factor subset that has been constructed, and the evaluation result is *^V^* = ^{*v*1,*v*2,*v*3,*v*4}^.

The membership function ^*R*_i_^ represents the degree to which each factor belongs to the evaluation result, and the method for determining ^*R*_i_^ is the trapezoidal curve method. Its mathematical expression is shown in Formula (12).


(12) $$G\,\left(F\,1,1.6,2.0,2.7\right)=\left\{ \begin{array}{c} \frac{1.6-F\,1}{2.0},F\,1<1.6\\ \frac{F\,1-1.6}{2.0-1.6},1.6<F\,1<2.0\\ \frac{F\,1-2.0}{2.7-2.0},2.0<F\,1<2.7\\ \frac{F\,1-2.7}{2.0}\,2.7<F\,1 \end{array}\right.$$

**FIGURE 3:**
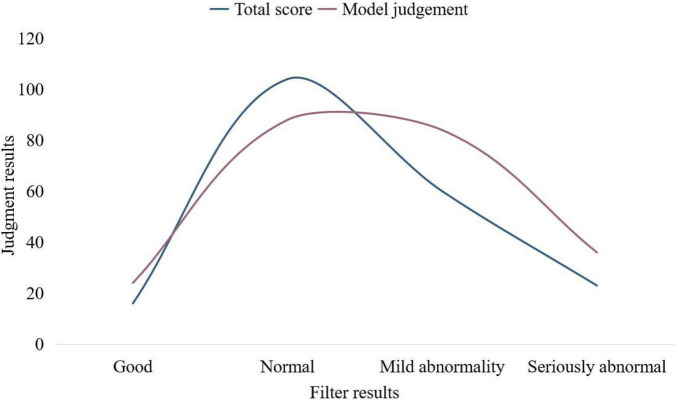
Comparison of screening results of fuzzy comprehensive evaluation.

Then, the membership degree of each factor in the second-level factor set is calculated according to the membership function. If ^*R*_i_^ is a single-factor matrix, the result is a first-level evaluation vector. The collected data were analyzed and integrated, and the results were obtained according to the total score system and model judgment, respectively, and the results of the SCL-90 scale screening were compared. The results are shown in Table 4 and Figure 3.

**TABLE 4:** Comparison results of data.

Serial number	Judgment value	Standard value	SCL-90 overall score
1	2	2	185
2	2	2	162
3	2	2	167
4	2	2	172
5	2	2	181
6	1	1	115
7	2	2	173
8	2	2	160
9	2	2	173
10	2	2	165

Due to the differences of poor university students, a new evaluation method is proposed in the screening method of the SCL-90 total score system. Taking the type of household registration, family composition, whether it is an only child, and 9 factors of the SCL-90 scale, a total of 12 factors are used as relevant factors to establish the factor set of the fuzzy comprehensive evaluation model for the psychological well-being status of poor university students, and the set-valued statistical iteration method is used. By calculating the membership degree, the membership degree of each factor obtained is more in line with the actual situation of poor university students. Therefore, the judgment model is more suitable for poor university students. Positive psychology believes that subjective positive psychological quality is how personal values are coordinated with the external world and the quality of life, and events that occur in individuals determine whether they can face and solve problems positively. Whether the individual’s satisfaction with the current life has reached the desired life index.

## Differential analysis of the influence of various variables on the psychological well-being of poor university students

### Analysis of the influencing factors of anxiety of poor university students

**FIGURE 4:**
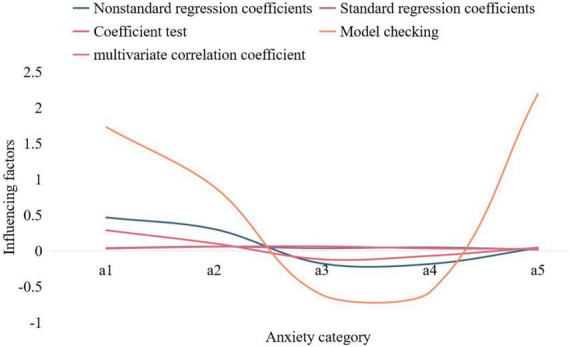
Influencing factors of anxiety of poor university student.

In this paper, self-esteem, family function, social support, and reasons for survival are used as independent variables, and the total anxiety score is the dependent variable with a = 0.07 as the inclusion standard, and multiple stepwise regression analysis is carried out. According to the standard regression coefficient, the factors are: al “self-esteem,” a2 “family function,” a3 “self-acceptance,” a4 “subjective support,” a5 “future optimism.” The multivariate correlation coefficient of the regression equation is 0.3800, and the coefficient of determination is 0.172, which is determined by the changes of the above five items. Other items had no significant effect and were excluded from the regression equation. The multiple regression equation is as shown in Formula (13).


(13) $$F\,i\,t\,\left(X\right)=\left\{ \begin{array}{c} C_{\max}-f\,\left(X\right),f\,\left(X\right)<C\\ C_{\min}+f\,\left(X\right),f\,\left(X\right)+C>C \end{array}\right.\,$$

where ^*C*_*min*_^ is the given smaller number and ^*C*_*max*_^ is the given larger number. According to the specific problem, choose the appropriate fitness function. The analysis of the influencing factors of the anxiety status of poor university students is shown in Figure 4.

The four indicators of depression, anxiety, life satisfaction and suicide risk of the university students who were poor were all worse than those of the university students without left-behind experience. Among them, from the results of the independent sample test, it can be seen that the four indicators of depression, anxiety, life satisfaction, and suicide risk of university students who were poor were all worse than those of university students who were not poor. Self-esteem, that is, the sense of self-worth, the affirmation of one’s own comprehensive value. Influenced by social comparisons, others’ evaluations, and self-affirmation of one’s own success or failure. The results of the independent sample test show that the self-esteem level of university students who have been poor is worse than that of university students without left-behind experience. At the same time, the regression analysis of psychological well-being influencing factors in this study shows that self-esteem has a strong predictability on the psychological well-being level of poor university students.

### Differences in psychological variables

**FIGURE 5:**
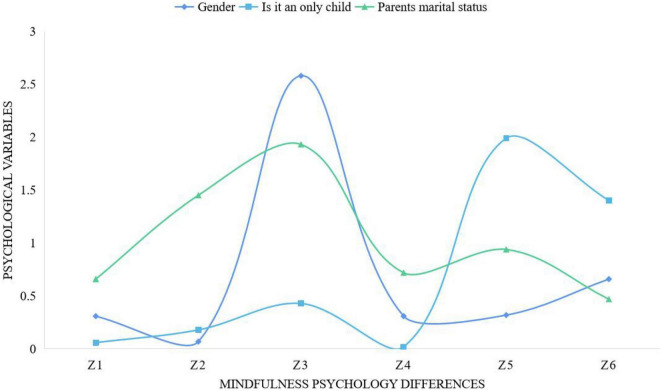
Psychological differences in mindfulness.

Mindfulness is a favorable means to improve psychological well-being, while rumination is an unfavorable factor affecting psychological well-being. There is a certain relationship between the two in terms of their connotations. Mindfulness can play a certain role in intervening in rumination. In order to analyze the total mindfulness score and the psychological differences of each dimension, such as whether there are gender differences in mindfulness scores, this study first performed a chi-square test on the distribution of psychological variables, and the results showed that there was no difference in the distribution of each variable. Then, a univariate difference test was carried out, and variables such as gender, whether it was an only child, and parental marital status were added to the fixed factor module in the general linear model to carry out multivariate analysis of variance. “Not responding,” “not judging,” “awareness and action,” and “total mindfulness score” are represented by Z1, Z2, Z3, Z4, Z5, Z6, respectively. The analysis results are shown in Figure 5.

From the results in Figure 5, it is found that when other psychological factors are considered, the differences in each dimension and total score of mindfulness are not significant. In addition, no interaction test was performed because the main effects were not significant.

**FIGURE 6:**
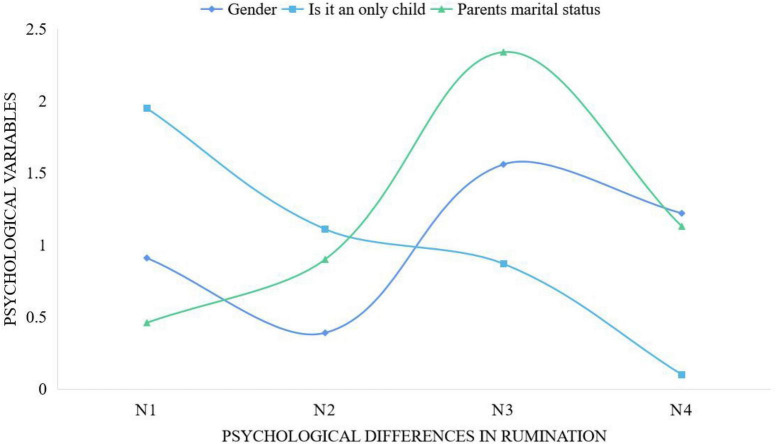
Psychological differences in rumination.

In order to analyze the total score of rumination and the psychological differences of each dimension, a multivariate analysis of variance was performed. The psychological differences of rumination are “symptom-related,” “compulsive thinking,” “reflective rumination,” and “ruling” are divided into N1, N2, N3, N4. It was found that all dimensions and total scores of rumination are in the psychological variables. There was no significant difference in the above analysis results, as shown in Figure 6.

**FIGURE 7:**
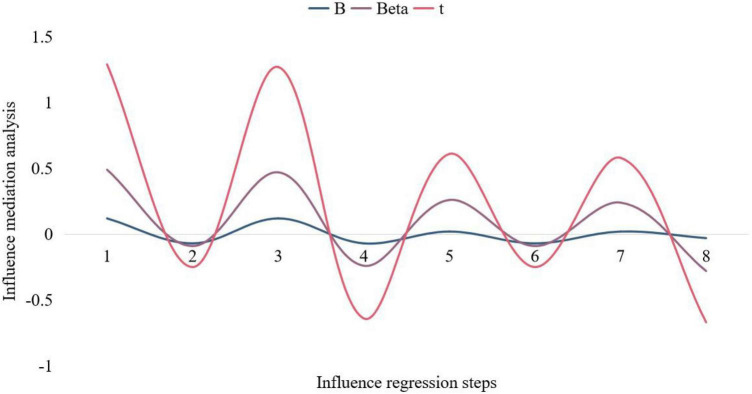
Mediation analysis of the effects of mindfulness.

**FIGURE 8:**
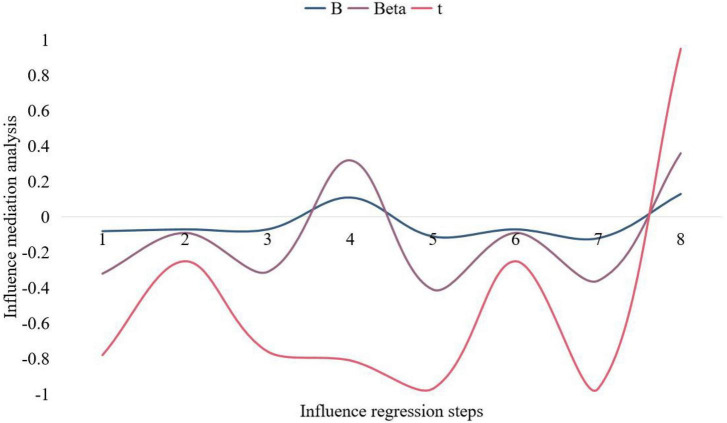
Mediation analysis of the effect of rumination.

In order to analyze the mediating effect of rumination on mindfulness and positive dimensions of psychological well-being (self-esteem and subjective well-being), this paper conducted a hierarchical regression analysis on mindfulness, rumination, and psychological well-being. The regression and the joint regression of mindfulness and rumination on self-esteem and subjective well-being all reached a significant level, it can be considered that rumination has a mediating effect in the influence of mindfulness on the negative dimension of psychological well-being, and since the addition of mindfulness after rumination has a mediating effect on anxiety and depression. People who ruminate will think about something repeatedly in their own minds, trying to find the right solution; Constantly thinking about the cause of the problem, the methods to solve the problem and weighing the direct benefits of these methods. When this situation becomes more and more out of control, the thinker will be trapped in a quagmire and develop into clinical depression. The regression is still significant, indicating that the mediating effect of rumination is a partial mediating effect. The analysis results are shown in Figures 7, 8.

The results show that the mediating effect of rumination is significant, but since this study also assumes that rumination has a moderating effect on mindfulness and psychological well-being, in order to comprehensively analyze the relationship between mindfulness, rumination and psychological well-being, this study is still strictly adjusted according to the procedure. Analysis of the effect. When mindfulness, rumination, and the product term of mindfulness and rumination are added as independent variables, the regression equations of self-esteem and subjective well-being are still significant, and the product term reaches a very significant level. The moderating effect of thinking on the positive dimension of psychological well-being is significant. Positive emotion is the key factor to ensure the healthy growth of university students. After correct emotional guidance, help university students develop good self-control and a positive side. Self-emotion management is the most effective way to control bad emotions. When students find that they have psychological problems, they should be able to control themselves, avoid the causes of negative emotions, and overcome psychological obstacles. At the same time, college students should also find their own ways to vent their pressure through communication or sports, and actively understand and learn some ways of music to regulate their emotions, so as to better help themselves out of the dilemma. Through venting room, exercise, listening to music, reading and other methods to relieve or eliminate negative emotions, consciously guide individuals to form an optimistic, confident and ideal state of mind. The regression model was used to analyze the relationship between mindfulness, rumination and psychological well-being. The mediation index fitted by the model reached 0.9. The model can reflect the real situation of the data, that is, rumination plays a partial mediating role in the effect of mindfulness on psychological well-being.

## Psychological well-being education strategies for poor university students based on differences in psychological prediction

The occurrence of psychological well-being problems is closely related to the individual’s non-acceptance of the self. Poor university students are more accustomed to focusing on their own shortcomings and defects. They compare their own shortcomings with the advantages of their classmates, and then “Discovering” how unbearable you are. While many underprivileged university students know that masochistic comparisons like this are useless for self-development, they automatically place their performance in the ranks of losers when they see their peers excel. Poor and poor university students The poor university students group is a very special group. They bear the expectations of their families and social responsibilities. Especially in the contemporary age when various undertakings in the motherland are flourishing, the pressure problem of poor university students is unprecedentedly prominent. In such an environment and background, many impoverished university students have very high requirements for themselves, and try their best to avoid mistakes that may occur in life and study. Once some negligence leads to results that are not as good as they imagined, they will blame themselves. Even sadness and depression appear, which seriously affects psychological well-being and normal learning. Therefore, it is particularly important to walk out of the shadow of the past life and no longer be affected by the past life. As stated in the results of this study, except that the “reflective meditation” dimension of rumination is not significantly related to the descriptive dimension of mindfulness, the negative correlation between the total score of mindfulness and its various dimensions and the total score of rumination and its various dimensions has reached a significant level. It can be considered that the higher the level of mindfulness, the lower the level of rumination. This is basically consistent with previous research results. If a college student can better focus on the present, accept and not evaluate, the individual will be less likely to be immersed in the negative events experienced in the past, that is, the chance of rumination is relatively less. Of course, relevant research cannot explain the causal relationship between rumination and mindfulness. Even if individuals with higher levels of mindfulness experience less rumination, it cannot be further inferred that mindfulness will effectively reduce the level of rumination. Therefore, it is necessary to carry out regression analysis on the two, and deeply analyze the relationship between mindfulness and rumination.

Poor university students are a special group. In view of the various psychological problems of poor university students, colleges and universities should fully improve the psychological crisis early warning mechanism for poor university students, build a system of psychological education, prevention, consultation, and crisis intervention; Teachers as the main body of the psychological crisis intervention professional team; build a psychological crisis intervention backbone team with counselors as the main body; set up a peer psychological crisis intervention auxiliary team with poor university students as the main body. By giving full play to the role of classroom teaching as the main channel in the psychological well-being education of poor university students, a series of school-level elective courses in psychology and health education are set up, various special activities on psychological well-being education are carried out, a website for psychological counseling is established, and psychological well-being aspects are compiled and published. Professional newspapers, actively expand the coverage of psychological well-being education, etc., and carry out publicity and promotion of psychological well-being education knowledge through multiple channels. Through the psychological testing platform, we can timely discover susceptible groups, and give appropriate care to special groups, so that the school’s psychological well-being education work is more effective, and the psychological well-being level of poor university students is improved.

## Conclusion

The innovation of this paper is to understand the relationship between mindfulness and rumination and the mental health of poor college students through predictive analysis, so as to provide reference for the mental health education strategies of poor college students. Introducing this psychological prediction model into the mental health education of poor college students will not only help to improve the educational concept and expand the educational approach. Based on BP neural network algorithm and fuzzy comprehensive evaluation model, this paper investigates the mental health development of this group by using the internationally recognized SCL-90 scale and questionnaire survey method. The model can reflect the real situation of the data, that is, rumination plays a partial intermediary role in the impact of mindfulness on psychological well-being. The introduction of this psychological prediction model into the mental health education of poor college students will not only help to improve the educational concept and expand the educational approach, but also help to achieve the goal of mental health education of poor college students, so as to promote the improvement of the psychological quality of poor college students. It is helpful to achieve the goal of mental health education for poor college students, so as to promote the improvement of poor college students’ psychological quality.

However, the situational assessment in this paper is proposed for the lack of written assessment methods. It refers to the design of activity scenes related to students’ study and life. There is no strict procedure for evaluation, so it has great flexibility. It is an activity in which all factors participate together, and it can adopt the mode of continuous evaluation and continuous feedback. It needs further research and analysis to comprehensively reflect the real situation of curriculum phenomena and students’ psychological changes.

## Data availability statement

The original contributions presented in the study are included in the article/supplementary material, further inquiries can be directed to the corresponding author.

## Ethics statement

Written informed consent was obtained from the individual(s) and minor(s)’ legal guardian/next of kin, for the publication of any potentially identifiable images or data included in this article.

## Author contributions

The author confirms being the sole contributor of this work and has approved it for publication.
